# Effect of Nurse-Led Intermittent Bladder Catheterization on Recurrence of Female Urethral Stricture following Urethral Dilatation: A Randomized Controlled Trial

**DOI:** 10.12669/pjms.42.5.12309

**Published:** 2026-05

**Authors:** Farah Naz, Samina Kausar, Jamshed Rahim, Shazia Taj

**Affiliations:** 1 Farah Naz, MSN Scholar. Department of Nursing, University of Health Sciences, Lahore, Pakistan; 2 Samina Kausar Professor & Head of Institute of Nursing, Department of Nursing, University of Health Sciences, Lahore, Pakistan; 3 Jamshed Rahim Associate Professor of Urology, Department of Urology, Sheikh Zayed Hospital, University, Lahore, Pakistan; 4 Shazia Taj, MSN Scholar.Department of Nursing, University of Health Sciences, Lahore, Pakistan

**Keywords:** Female urethral stricture, Intermittent bladder catheterization, Urethral dilation, Nurse-led intervention

## Abstract

**Background & Objective:**

Female urethral stricture (FUS) is a rare but significant cause of bladder outlet obstruction, often leading to urinary retention, recurrent UTIs and other complications. Conventional treatment like urethral dilation provides temporary relief but has a high recurrence rate. Intermittent bladder catheterization (IBC) is a promising adjunct following dilation that may reduce recurrence by maintaining urethral patency and preserving bladder health. Despite its effectiveness in managing urethral strictures, there is limited evidence regarding its implementation in female populations particularly as a nursing intervention. This study aimed to evaluate the impact of intermittent bladder catheterization (IBC) on reducing the recurrence of urethral strictures in female patients following urethral dilation.

**Methodology:**

A randomized controlled trial was conducted at the Urology Department of Shaikh Zayed Hospital, Lahore, from May 2024 to December 2024. A total of 56 female patients (aged 35-55 years) were recruited and randomized into two groups: the intervention group (n=28), which performed IBC twice daily at home under nursing supervision, and the control group (n=28), which received standard post-dilation follow-up without IBC. Stricture recurrence was assessed over a follow up of 12 weeks through periodic catheterization using a 14Fr Nelton catheter. Relative risk and absolute risk of recurrence reduction was calculated. Confidence interval of 95% was calculated for all estimates.

**Results:**

For data analysis, Chi-square test, independent t-test and repeated measure ANOVA were used. The recurrence rate was significantly lower in the intervention group compared to control (7.14% vs 28.57%, p = 0.03). The relative risk of recurrence was 0.25 (95%CI: 0.06–0.98), indicating a 75% reduction in recurrence with intermittent bladder catheterization.

**Conclusion:**

Nurse-led intermittent bladder catheterization (IBC) after urethral dilation, is an effective strategy in reducing stricture recurrence among the females.

***Clinical Trials Number:*** ID NCT06064968.

## INTRODUCTION

Female urethral stricture (FUS) is anatomical narrowing of distal urethral lumen less than 14Fr which doesn’t accommodate any instrumentation without affecting urethral mucosa.^1^ It is usually suspected in a patients with urinary retention following unsuccessful attempt at catheterization per-urethra, confirmed subsequently on urethroscopy.^2^ Women who present with lower urinary tract symptoms like frequency, urgency, nocturia, straining on voiding, weak flow of urine, intermittency, incomplete emptying; have 2.7%-8% chances of having bladder outlet obstruction (BOO), and out of these 4%-13% females have urethral stricture.^3^ In United States nearly about 200 million dollars are spent on urethral strictures where almost 5000 new patients are registered annually.^4^

Factors such as lack of definitive diagnostic criteria, very few (0.1 to 1%) females with voiding symptoms presenting to health care facilities for treatment and low index of suspicion for female urethral stricture disease and paucity of any targeted approach towards the management; all in turn lead to scarcity of data on the disease prevalence and its management.^5^ Likewise in an Indian study 6.8% incidence of urethral stricture has been reported.^6^

Urethral dilation has good initial symptomatic relief but to high recurrence rate, so intermittent bladder catheterization (IBC) is a favored to limit urethral stricture recurrence.^7^ It also maintains the bladder health, preserves renal function and prevents urinary tract infections in comparison to permanent indwelling catheter.^8^ IBC can be repetitively performed by patients themselves with great safety, minimizing the need for urethral reconstruction.^9^ In United Kingdom alone around 50,000 people per day perform intermittent bladder catheterization emptying bladder.^10^ IBC is one of those interventions which can be demonstrated and taught to the patients by a trained nurse.^11^

Despite the growing evidence supporting intermittent bladder catheterization following urethral dilation to reduce the urethral stricture recurrence, data on nurse-led implementation of intermittent bladder catheterization for reducing recurring stricture in female populations remains extremely limited, particularly in developing countries. In this study we recruited females having urethral stricture after they were treated with urethral dilation by the urologist and were advised post-operative intermittent bladder catheterization. IBC was taught and supervised by a trained nurse to study the effects of this intervention on reduction of stricture recurrence.

## METHODOLOGY

This randomized control trial was conducted in Urology Department of Shaikh Zayed Hospital, Lahore, Punjab, Pakistan in collaboration with Institute of Nursing, University of Health Sciences, Lahore, Pakistan. The study was conducted from May 08, 2024 to December 10, 2024. The study was registered at clinicaltrials.gov with unique ID NCT06064968.

### Ethical Approval:

It was approved by Institutional Review and Research Advisory Board (IRRAB) / Technical & Ethical Review Committee (TERC), vide letter no. TERC/NHRC-Internal-2/398, dated: December 16, 2023.

**Fig.1:**
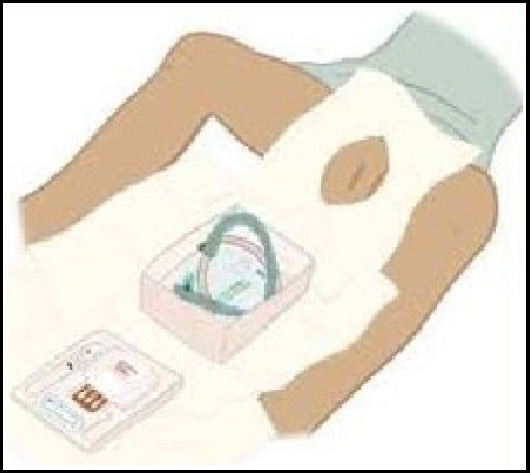
Frog leg position for performing IBC.

Following urethral dilation by the urologist for their urethral stricture disease, females between 35 to 55 years who required intermittent bladder catheterization were included. Their body mass index (BMI) was less than 30 and abbreviated mental test score (AMTS) was seven and above. Patients with obesity, physical disability and/or cognitive impairments were excluded from the study. A total of n=56 females were selected by convenience sampling because of low reported incidence of disease and less frequent presentation of the patients in health care facilities. This was followed by their randomization in control and intervention groups via lottery method by an independent staff member not involved in the study. Allocation concealment was ensured using sealed opaque envelopes. Due to the nature of the intervention, blinding of participants and outcome assessors was not feasible. In the control group (n=28), patients received standard follow up care without any intermittent catheterization at home. However, in the intervention group (n=28), patients performed intermittent bladder catheterization themselves twice daily at home i.e. first one early in the morning just before first void and the second at night before going to bed. All the participants of the group were individually counseled about the procedure and properly trained by the dedicated urology nurse with practical demonstration of the technique in different positions like lying supine with frog leg position (Fig.1) and in crouching position just like using an Indian toilet seat. It was made sure that all patients completely understood the procedure and have become trained enough to perform IBC at home after training.

**Fig.2:**
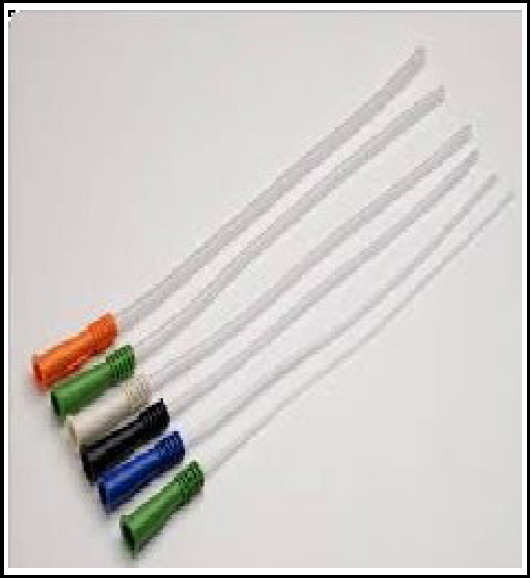
Nelton catheters of various sizes.

A clean non-sterile technique for catheter insertion was used with Nelton catheter of size 14Fr (3French= 1mm) (Fig.2) along with a topical anesthetic jelly (lignocaine gel 2%). After instillation into urethra, the jelly was also applied on the catheter tip for its smooth insertion through the urethra into (a preferably filled) bladder till clear urine started draining out with subsequent gentle removal of Nelton catheter.

Patients in both groups were followed after every two weeks for a period of 12 weeks and were assessed by the urology nurse for stricture recurrence by passing 14Fr Nelton catheter through urethra into the bladder on each visit. No imaging and/or uroflowmetry was used for stricture assessment as it was beyond the nursing domain. Successful catheterization ruled out urethral stricture recurrence and the patients were scheduled for the next follow up after 15 days. On the contrary, failure to catheterize at any of the follow up visits indicated urethral narrowing and labelled as stricture recurrence. These patients were excluded from the subsequent follow ups and referred back to the urologist.

## RESULTS

Demographic details of the patients were demonstrated in terms of frequencies and percentages. Mean and standard deviation were also calculated. Chi Square was applied to assess difference between the groups and an independent *t-test* applied to assess the effect of Intermittent Bladder Catheterization (IBC) on preventing the urethral stricture recurrence. Data were analyzed using SPSS version 23. Categorical variables were compared using the Chi-square test. Relative risk (RR) and odds ratio (OR) with 95% confidence intervals were calculated to determine the effect size. A p-value of ≤0.05 was considered statistically significant.

In intervention group, age range was 28 to 52 years with a mean of 42.96 ± 6.75 years whereas in control group, age range was 45 to 55 years with a mean of 49.39 ± 3.41years. Weight range of the patients were similar in two groups i.e. 40Kg to 50 Kg with mean of 45 ± 2.75 Kg and 40Kg to 52Kg with mean of 46.3 ± 3.00 Kg in intervention and control groups respectively. Overall, both groups revealed comparable BMI, with the control group showing a slightly higher average and dispersion as compared to intervention group (i.e. 27.46 ± 1.16 vs. 27.28 ± 0.95 respectively).

Overall recurrence in both groups was 10/56 (17.86 %). A significant difference in recurrence rates was observed between the two groups (p = 0.03) (Table-II). The recurrence rate was lower in the intervention group 2/28 (7.14%) compared to the control group 8/28 (28.57%). The relative risk (RR) of recurrence was 0.25 (95% CI: 0.06–0.98), indicating a 75% reduction in risk among patients receiving intermittent bladder catheterization. The odds ratio (OR) was 0.19 (95% CI: 0.03–0.99), demonstrating a strong protective effect of the intervention. The absolute risk reduction was 21.43%, corresponding to a number needed to treat of 5, indicating that five patients need to receive the intervention to prevent one recurrence.

**Table-I:** Baseline Characteristics of Participants.

Parameters	Groups	N	Minimum	Maximum	Mean	Standard Deviation
Age	Intervention	28	28 years	52 yeas	42.96 years	6.75
	Control	28	45 years	55 years	49.39 years	3.41
Weight	Intervention	28	40 Kg	50 Kg	45.0357 Kg	2.75523
	Control	28	40 Kg	52 Kg	46.3571 Kg	3.00881
Height	Intervention	28	1.5 m	1.83 m	1.6514 m	.09602
	Control	28	1.52 m	1.88 m	1.6889 m	.11380
Body mass index (BMI)	Intervention	28	25.68 Kg/m^2^	28.75 Kg/m^2^	27.2779 Kg/m^2^	.94751
	Control	28	24.70 Kg/m^2^	28.90 Kg/m^2^	27.4682 Kg/m^2^	1.16543

**Table-II:** Comparison of “Urethral Strictures Recurrence” between Groups.

Variable	Recurrence n (%)	Recurrence n (%)	Total	p-value	Relative Risk (95% CI)	Odds Ratio (95% CI)
	Yes	No				
Control Group	8 (28.57%)	20 (71.43%)	28	0.03	0.25 (0.06–0.98)	0.19 (0.03–0.99)
Intervention Group (IBC)	2 (7.14%)	26 (92.86%)	28			

These results suggest that intervention in terms of intermittent bladder catheterization (IBC) was effective in reducing the urethral stricture recurrence when compared to the control group.

## DISCUSSION

This study included treated cases of urethral strictures with females with between 35 years to 65 years with a mean of 42.96 ± 6.75 in intervention group and 49.39 ± 3.41 in control group. These figures correspond to a study on female population with urethral stricture disease with a mean age of 49 years with age range of 25-75years.^6^ Our recruits were generally within the overweight range according to World Health Organization general classification.^12^ The intervention group having BMI 27.28 ± 0.95 kg/m^2^ and control group showing 27.47 ± 1.17 kg/m^2^, which are close to the mean BMI of 29 kg/m^2^ reported in a study.^13^ In the females of this age range there was high likelihood of finding definite cases of urethral stricture disease where cognition and functional capacity (assessed via AMTS score in our study) is preserved along with dexterity needed to perform IBC of the recruits which is possible with this BMI range. All the above factors reportedly have been associated with the success of the study.^14^

A total of 56 female patients with a diagnosis urethral stricture were randomized into intervention and control groups having 28 patients in each group. In published data worldwide, however, rather lower numbers of female participants in different studies have been reported i.e., 7- 82 patients with variety of diagnoses ranging from meatal stenosis to bladder neck contractures to functional urethral syndrome.^6^ None of these studies focused on the female urethral stricture exclusively in contrast to our study. However, some researchers did recruit only the confirmed cases despite the low prevalence (2.7% to 23%) and controversies in the standardizing the diagnosing criteria for female urethral stricture disease.^6^ However, in contrast to female urethral stricture, male urethral stricture disease has been reported to have more definite diagnostic criteria, hence a much higher incidence (0.6% - 0.9%) has been presented in various studies.^15^,^16^

The 14Fr size urethral catheter was used for intermittent bladder catheterization (IBC) in our study and literature also supports the use of a wide range of catheter size i.e. from as small as a 5Fr catheter to as large as a 22Fr, for intermittent bladder catheter (IBC) to maintain urethral patency.^17^

Intermittent bladder catheterization was performed by the patients in intervention group twice a day for 12 weeks after training by the urology nurse. Gray M. et al in their study had also advocated intermittent bladder catheterization under nursing supervison.^11^ However, in our health care system, stereotypically doctors have been reported to teach the patient how to perform intermittent catheterization using nelton catheters.^18^,^19^

A wide range of durable results after urethral dilation alone (i.e. 0-67%) haven been reported so as for the ‘on demand urethral dilation’ after patient had presented with urethral stricture recurrence with better success rates post procedure.^5^ Our study (urethral dilation with IBC), however, reported an overall success rate of 82.14% with a recurrence rate of only 17.86% with significant difference of reported recurrence between the control group i.e. eight cases (28.57 %) versus intervention group with two cases (7.14%) in 12 weeks of follow up. Due to lack of studies on female urethral stricture disease such results cannot be compared within the local population. However, a similar result with 81.39% recurrence free population has been reported in male urethral stricture disease following endoscopic urethral stricture treatment and intermittent catheterization in males.^18^

Furthermore, a much higher recurrence rates have been reported in a study when a group wise comparison with our study was assessed in intervention (22% vs. 7.14%) and control (46% vs. 28.57%) groups.^19^ In a study conducted in India on males described that intervention group performing intermittent catheterization after endoscopic treatment of urethral stricture disease poses significant impact on recurrence (i.e., 20%) in contrast to that of recurrence in control group (75%).^20^

Out of all the recurrences in both groups combined (n=10) in our study, the timing of every individual case in each group was also analyzed. It was noticed that majority (7 /10) of these recurrences appear on first follow up after two weeks’ time with five cases in ‘control group’ and two cases in ‘intervention group’. Rest of the three cases with urethral stricture recurrence were reported in ‘control group’ only with two cases at the third follow-up and one participant at the fourth follow-up. Although similar recurrence rates have been mentioned within a certain follow up time ranging from eight weeks to one year, however, none of these shows specifically the time of recurrence of individual case.^18^,^19^

### Limitation:

Unknown disease incidence in local population, single center study, a handful of study recruits, selection bias, limited duration of study, shorter follow up time, less data on females for comparison are the limitations of this study.

### Recommendations:

A much larger population sample with longer duration and preferable a multi-centric study on urethral stricture disease and recurrence is needed to be designed for over generalizing the role of a nurse-led intermittent bladder catheterization.

## CONCLUSION

Nurse-led intermittent bladder catheterization following urethral dilatation significantly reduces short-term recurrence of female urethral stricture and represents a practical, patient-centered, and cost-effective intervention by replacing the need for specialist doctor for caring such patients.
